# Recommendations for dietary level of micro-minerals and vitamin D_3_ to Atlantic salmon (*Salmo salar*) parr and post-smolt when fed low fish meal diets

**DOI:** 10.7717/peerj.6996

**Published:** 2019-05-31

**Authors:** P. Antony Jesu Prabhu, Erik-Jan Lock, Gro-Ingunn Hemre, Kristin Hamre, Marit Espe, Pål A. Olsvik, Joana Silva, Ann-Cecilie Hansen, Johan Johansen, Nini H. Sissener, Rune Waagbø

**Affiliations:** 1 Institute of Marine Research, Bergen, Norway; 2 Faculty of Biosciences and Aquaculture, Nord University, Bodø, Norway; 3 Biomar AS, Trondheim, Norway; 4 Norwegian Food Safety Authority, Oslo, Norway; 5 GIFAS, Salten Havbruksparken, Sundsfjord, Norway

**Keywords:** Zinc, Selenium, Micro-minerals, Parr, Post-smolt, Salmonids, Vitamin D3, Bone metabolism

## Abstract

Atlantic salmon (*Salmo salar*) feeds have changed drastically in their composition from being predominantly marine-based to plant-based. This has altered the dietary supply and availability of micro-nutrients to Atlantic salmon. The impact of graded inclusion levels of a nutrient package (NP) comprising of 25 different micro-nutrients were studied in Atlantic salmon parr in freshwater (Trial 1) and post-smolts in seawater (Trial 2). In brief, the NP was included from 0 to 400%, where 100% corresponded to the recommendation by the National Research Council, 2011. Micro-nutrients, namely Zn, Mn, Se, Cu, Fe, Co, I and vitamin D3 were included in the NP with the objective of (re)evaluating the dietary need to meet the requirement of Atlantic salmon parr and post-smolt, when fed low fish meal, plant ingredient-based diets. Responses in apparent availability coefficient (AAC), whole body and vertebrae mineral concentrations, and retention were analysed. AAC of Cu, Mn, Se and Zn responded in a quadratic fashion with an increase in NP from 0 to 400% in freshwater parr; AAC could not be measured in post-smolt salmon. The whole-body concentration of Zn, Se, Co and I in Atlantic salmon parr were significantly affected by increasing NP inclusion; the same was observed for Zn, Se and Co in post-smolt Atlantic salmon. Vertebrae mineral concentration as the response criterion was non-responsive in parr; whereas, in post-smolt, Co had a linear increase, while Zn and Se showed a non-linear increase upon 0 to 400 NP inclusion. Zinc concentration and activities of alkaline phosphatase (ALP) and tartrate-resistant acid phosphatase (TRAP) in vertebrae indicated increased bone resorption in post-smolt Atlantic salmon; TRAP activity increased linearly with NP inclusion in post-smolt, but not in parr. Significant correlations between Zn and Se were observed in AAC and vertebral concentrations, indicating an interaction in intestinal uptake and vertebral deposition. Overall, Atlantic salmon parr held in freshwater were able to satisfy the requirement for the trace minerals Zn, Mn, Se, Cu, and Fe through supply from 100–150 NP, corresponding to 101–132, 47–63, 0.6–0.8, 12–16 and 150–166 mg kg^ −1^, respectively; for iodine, dietary supply from 150–200 NP, corresponding to 0.7–1.6 mg kg^−1^, was required. In the seawater, Atlantic salmon post-smolt, in general, required micro-minerals and vitamin D3 levels as supplied through 150–200 NP, corresponding to 140–177, Zn; 61–67, Mn; 0.9–1, Se; 14–16, Cu; and vitamin D3, 0.06–0.09 mg kg^ −1^ to fulfil the requirement, except for Cu which was satisfied at 100–150 NP, equivalent to 13–14 mg kg^ −1^ diet.

## Introduction

Sustainable growth of Atlantic salmon farming is faced with many challenges related to fish welfare and environmental impacts (Olesen, Myhr & Rosendal, 2011). Production-related disorders is a genuine concern in Atlantic salmon farming and are often linked to dietary micro-nutrient supply (Baeverfjord et al., in press). On one hand, sufficient dietary supply of micro-nutrients vital for proper bone health and metabolism are important to ensure fish welfare. On the other hand, it is also equally important to limit the environmental load of minerals, by avoiding excess dietary supply. Atlantic salmon (*Salmo salar*) feeds have changed drastically in their composition from being predominantly marine based in the 1990s to plant-based at present, especially as a protein source (Ytrestoyl, Aas & AAsgaard, 2015). Ensuring the balance of limiting nutrients like histidine, taurine, and essential fatty acids (arachidonic acid, ARA; eicosapentaenoic acid, EPA; docosahexaenoic acid, DHA) have enabled better growth, health and production when fed diets with high inclusion of plant ingredients. However, efforts to understand the impact of the change in diet composition on micronutrients have largely been ignored, until recently. Minerals have structural importance and functional roles as part of metalloenzymes and metalloproteins; the macro-minerals like phosphorus, calcium and magnesium are major structural components of bones and hard tissues (Lall & Lewis-McCrea, 2007), while potassium and sodium facilitate osmoregulation, acid–base balance and cellular nutrient transport (Kaushik, 2002). The micro-minerals, such as copper (Cu), cobalt (Co), iron (Fe), iodine (I), manganese (Mn), selenium (Se) and zinc (Zn) have important functional roles to play in hepatic and bone metabolism, anti-oxidant and redox regulation, thyroid metabolism and innate immunity (Watanabe, Kiron & Satoh, 1997; Lall, 2002). Along with minerals, vitamin D is important for regulating mineral and bone metabolism (Lall & Lewis-McCrea, 2007).

Structurally, bones are the major storage site for Zn and Mn; Zn, Mn and Cu are also integral parts of the enzyme cascade involved in bone remodeling. Alkaline phosphatase was the earliest discovered Zn containing protein; while Zn, Cu and Mn are structural components of superoxide dismutase; Se imparts its role in bone remodeling through glutathione peroxidase. A more detailed account of minerals in salmonid bone metabolism and health can be found in a recent review (Baeverfjord et al., in press). Vitamin D is of significance in regulating mineral utilization and has an important role to play in bone metabolism and remodeling (Lock et al., 2010). National Research Council’s (NRC) recommendations are kept as a reference with regard to establishing dietary nutrient levels in fish feeds. Unfortunately, the NRC had reviewed the recommendations only once in 2011 since the inaugural edition on fish in 1990. The NRC values on nutrient requirements are obtained from studies using highly purified ingredients, therefore the values presented represent near 100 percent bioavailability (NRC, 2011). However, in commercial feeds made of practical ingredients, the presence of multi-nutrient interactions, anti-nutritional factors and even the feed processing conditions interfere with the availability of micro-nutrients. Therefore, it is essential to define ‘practical recommendations’ for micro-nutrients in salmonid feeds.

Fish meal has generally been regarded as a good source of micro-nutrients in fish feeds (Julshamn, Haugsnes & Utne, 1978; Lorentzen & Maage, 1999). Over the years, the inclusion of plant ingredients in the place of fish meal has increased steadily, concurrently changing the micro-nutrient profile of the feeds. Moreover, plant ingredients are known to contain one or more anti-nutritional factors, which further limits the availability of inherent and supplemented micro-nutrients. Recent reports show that the available levels required for Zn, Mn and Se in complete plant-based diets for rainbow trout to be higher than recommended by NRC (2011) (Welker et al., 2016; Antony Jesu Prabhu et al., 2018a; Welker et al., 2018). Life stage and environment are other factors that might also alter dietary nutrient requirements in fish, especially of minerals which can also be absorbed from the water (Bury, Walker & Glover, 2003). Atlantic salmon, being an anadromous fish, spends the initial stage of the life cycle in freshwater rivers (parr) and then upon smoltification, moves to saltwater in the sea (post-smolt). To date, most of the micro-nutrient requirement estimates available for Atlantic salmon pertain to parr in freshwater (NRC, 2011). Very little is known of the micro-nutrient requirements of the post-smolt in seawater, should there be a difference between the two life stages.

In the present study, a regression design was adopted to test graded inclusion of a micro-nutrient package (NP) consisting of 25 different micro-nutrients in low fish meal Atlanitc salmon feeds, both in freshwater and seawater (Hamre et al., 2016; Hemre et al., 2016a). The micro-nutrient mix was added to the feeds from 0 to 400% NP, where 100% NP represented the NRC (2011) recommended levels. The outcome of this study was split into three scientific articles based on micro-nutrient classes and their major functions. In our two earlier reports, updated recommendations with regard to amino acids and B-vitamins related to nutrient and energy metabolism (Hemre et al., 2016a); vitamin C, vitamin E and selenium, the micro-nutrients related to redox regulation were presented (Hamre et al., 2016). This paper represents the third of the series and presents the findings on dietary levels required for selected micro-minerals and vitamin D3 related to micro-mineral balance and bone metabolism in Atlantic salmon fed plant-based diets. The combination of 25 micro-nutrients together as experimental variables does make this design less appropriate to eliminate the different micro-nutrient interactions, nevertheless the outcome has relevant scientific merit (Hamre et al., 2016; Hemre et al., 2016a) and practical implications (Hemre et al., 2016b). Therefore, this study aimed to (i) define new recommendations for dietary levels of the different micro-nutrients as a nutrient package (NP) to Atlantic salmon when fed diets with high plant ingredients, and to (ii) identify if the NP inclusion level varied with the life stage between parr in freshwater and post-smolt in seawater.

## Materials & Methods

### Experimental design and diets

As the data presented in this manuscript was generated from the same feeding trials as previously described (Hamre et al., 2016; Hemre et al., 2016a), experimental set-up and the test diets (produced at Biomar Technology Centre, Denmark) were the same. In brief, this study in Atlantic salmon was part of the larger EU-funded Arraina project (http://www.arraina.eu), aimed to establish updated micro-nutrient recommendations for five major aquaculture fish species in the EU, including Atlantic salmon, when fed diets with high inclusion of plant ingredients. The study followed a regression design with graded inclusion levels of a multi-nutrient premix (nutrient package, NP with 25 nutrients) in two trials (i) Atlantic salmon parr in freshwater (Trial 1), and (ii) Atlantic salmon post-smolt in seawater (Trial 2). Both trials were designed in a dose–response manner with the exact same diet-design (apart from the adjustment of protein and lipid levels according to fish size), using seven diets with graded levels of an NP added to a basal diet. The NP contained essential vitamins, minerals, cholesterol and amino acids (totally 25 nutrients), where the 100% inclusion level (100 NP) was close to NRC (2011) recommendations for salmonids (mainly rainbow trout). The inclusion of the NP ranged from no addition to four-fold the 100% addition (0 NP, 25 NP, 50 NP, 100 NP, 150 NP, 200 NP and 400 NP). The feed formulation and analyzed nutrient composition of the feeds are provided in Tables 1 and 2, respectively.

**Table 1:** Feed formulation for Atlantic salmon parr in Trial 1, and with slight difference made for post-smolt in Trial 2 given in parentheses. Nutrient package (NP), histidine and cholesterol were added to the diets in graded amounts and balanced by reducing the content of field peas in the diets, all other ingredients were used in equal amounts in all diets. Units are in g kg^−1^.

**Composition**	**0 NP**	**25 NP**	**50 NP**	**100 NP**	**150 NP**	**200 NP**	**400 NP**
Fish meal SA 68 superprime	80	80	80	80	80	80	80
Krill meal	24.2	24.2	24.2	24.2	24.2	24.2	24.2
Soy Prot. Conc. 60%	180	180	180	180	180	180	180
Corn gluten 60	40	40	40	40	40	40	40
Pea protein 75	124 (130)	124 (130)	124 (130)	124 (130)	124 (130)	124 (130)	124 (130)
Wheat gluten	180 (150)	180 (150)	180 (150)	180 (150)	180 (150)	180 (150)	180 (150)
Wheat	61 (60)	61 (60)	61 (60)	61 (60)	61 (60)	61 (60)	61 (60)
Field peas	100	98	95	90	85	80	60
Fish oil, capelin	35 (44)	35 (44)	35 (44)	35 (44)	35 (44)	35 (44)	35 (44)
Rapeseed oil	79 (88)	79 (88)	79 (88)	79 (88)	79 (88)	79 (88)	79 (88)
Linseed oil	22	22	22	22	22	22	22
Palm kernel oil	44 (48)	44 (48)	44 (48)	44 (48)	44 (48)	44 (48)	44 (48)
**Nutrient premix^a^**	**0**	**0.25**	**0.5**	**1.0**	**1.5**	**2.0**	**4.0**
Histidine	0.00	0.34	0.68	1.36	2.04	2.72	5.44
Cholesterol	0.00	0.28	0.56	1.13	1.69	2.25	4.50

**Notes.**

a Factor of requirement recommended by NRC (2011). All diets were added 38 g kg monosodium phosphate, 9.3 g kg lysine, 1.8 g kg^−1^ threonine, 8 g kg^−1^ choline (50%), 0.25 g kg^−1^ barox becp dry, 0.5 g kg^−1^ yttrium oxide. The 100 NP contained (in ppm) minerals, (selenium, 0.23; iodine, 0.67; copper, 3.2; cobalt, 0.94; manganese, 12; iron, 32.6; and zinc, 67); vitamins (vitamin D3, 0.05; *α*- tocopherol-acetate, 102; vitamin K3, 9.8; vitamin A1, 3.8; ascorbyl monophosphate, vitamin B6, 4.8; biotin, 0.14; cobalamin, 0.25; folate, 2.8; pantothenic acid, 17.2; riboflavin, 8.3; thiamine, 2.7; and niacin, 24.8), crystalline DL-methionine, 510; and taurine, 2450. The mineral sources used were all inorganic: Se, sodium selenite; Zn, zinc oxide; Mn, Manganous oxide; Cu, Cupric sulphate pentahydrate; I, Calcium iodate anhydrous and Fe, ferrous sulphate monohydrate; Cholecalciferol was used as the source of vitamin D3.

**Table 2:** Analyzed proximate and nutrient composition of feeds in trial 1 (parr) and trial 2 (post- smolt). Values provided in parentheses pertain to the concentration of the respective nutrient in the feeds used in Trial 2. All results are the mean of two analytical parallels. Protein, lipid, starch ash and dry matter are given in g kg^−1^, energy in MJ kg^−1^, while all other diet components are given as mg kg^−1^.

	**0 NP**	**25 NP**	**50 NP**	**100 NP**	**150 NP**	**200 NP**	**400 NP**	**Requirement**^e^
*Proximate composition, g kg*^−1^								
Dry matter	910 (950)	930 (940)	920 (930)	920 (930)	930	920 (930)	920	–
Energy, MJ kg^−1^	22.8	22.7	22.6	22.7	22.4	22.5	22.0	–
Protein	453 (480)	469 (472)	449 (440)	456 (480)	462 (480)	470 (480)	461 (480)	360 (digestible)
Lipid	213 (220)	203 (220)	219 (210)	211(230)	208 (220)	197 (240)	195 (220)	–
Starch	112	112	109	104	106	107	94	–
Ash	66	68	66	67	69	60	75	–
Phosphorus^a^	14.0	–	–	–	–	–	–	7 (available)
Calcium^a^	4.4	–	–	–	–	–	–	NR
*Micronutrients supplemented at graded level, mg kg*^−1^								
Vitamin D3	<0.01^b^	0.01	0.02	0.04	0.06 (0.09)	0.09 (0.1)	0.15 (0.14)	0.04 (R.T)
Cobalt	0.37 (0.17)	0.33 (0.36)	0.53 (0.65)	1.04 (1.2)	1.71 (1.7)	2.15 (2.3)	3.65 (2.9)	–
Copper	8.31 (9.4)	8.91 (11.4)	10.2 (11.7)	12.7 (13.4)	16.4 (14.3)	20.4 (15.8)	30.0 (21.3)	5
Iodine^c^	0.13 (0.28)	0.11 (0.46)	0.23 (0.85)	0.41 (1.23)	0.70 (1.46)	1.58 (1.39)	2.74 (2.31)	1.1/1 (R.T./P.S.)
Iron^d^	117 (424)	126 (375)	134 (396)	150 (424)	166 (414)	183 (433)	248 (516)	30–60
Manganese	37.1 (36.3)	41.1 (35.6)	41.9 (40.9)	47.1 (50.7)	63.3 (61.4)	60.2 (66.7)	95.8 (85.2)	10
Selenium	0.42 (0.47)	0.45 (0.48)	0.52 (0.56)	0.62 (0.79)	0.80 (0.91)	1.04 (1.04)	1.39 (1.1)	0.15 (R.T)
Zinc	62.0 (57.5)	66.9 (66.9)	76.9 (88.7)	101 (115)	132 (140)	159 (177)	243 (191)	37

**Notes.**

NP nutrient package

a Analysed only in the 0 NP diet.

b Below the limit of quantification, uncertain value.

c Large variation between analytical parallels was seen for iodine, including after re-analysis of samples.

d Due to a mistake, Fe was not analyzed in the feeds, thus nominal values are used in the table.

e Requirement according to (NRC, 2011), in cases where requirement for Atlantic salmon was not listed, data for rainbow trout (abbreviated R.T.) and/or Pacific salmon (P.S.) are indicated in the table.

### Feeding trials

The two feeding trials were conducted in accordance with Norwegian laws and regulations concerning experiments with live animals, which are overseen by the Norwegian Food Safety Authority. Permissions to conduct the two experiments were given by the Directorate of Fisheries, and accepted for feeding trials at GIFAS, §13 (Akvakulturloven) and §28a (Lakseforskriften) (ref 13/11363) and acknowledged by the advisory board 27.11.12 (ref: ARRAINA regression trial permission).

Trial 1: Atlantic salmon parr (mean initial weight, of 18.3 ± 2.2 g) were randomly distributed in fifteen 400 L tanks (100 fish per tank), on a flow-through system (flow rate, 20 L min^−1^) at Matre experimental research station, Institute of Marine Research. The experimental feeding commenced on 3rd of July 2012 and continued for 12 weeks, with duplicate tanks for each diet, except for NP 100 that was run in triplicate. The fish were fed *ad libitum* with continuous feeding from automated feeders under a 24 h light regime (continuous light) and oxygen saturation was maintained above 75%. The fish were reared in freshwater, but with seawater added as a buffer, creating a salinity of 1.1–1.3 g L^−1^. The temperature was maintained at 12.4 ± 0.7  °C (mean ± SD) during the experimental period.

Trial 2: Atlantic salmon post-smolt (mean initial weight, 228 ± 5 g) were randomly distributed among fifteen sea cages (125 m^3^; 150 fish per cage) at Gildeskål Research Station, GIFAS, Gildeskål kommune, Norway. Prior to the start of the trial, fish were acclimated to the environmental conditions for two weeks; the experimental feeding started on Jan 2013. As in the freshwater trial, the dietary treatments were performed in duplicate cages, but for 100 NP in triplicate cages. The trial lasted for 157 days, under 24 h light regime before the start of the trial and during the first three months of the experiment and hand-fed to satiation twice a day. Mortality was recorded daily. Water temperature, salinity and oxygen saturation over the course of the trial varied from 4.1 (January) to 10 °C (June), 30-34.2 g L^−1^, and 8.7–12.2 mg L^−1^, respectively.

### Sampling

Fish were anesthetized (Benzoak R VET, 0.2 ml/L, ACD Pharmaceuticals, Leknes, Norway) and killed by a blow to the head. The general sampling procedures followed thereafter are detailed in the earlier report (Hemre et al., 2016a). The sampling procedures only pertaining to the data presented in this manuscript are described here. At the end of both trials, 10 fish per cage were homogenized to collect samples for whole-body mineral composition analyses. Vertebrae samples (removed for neural and hemal arches and adhering skeletal tissue) from 10 fish for each cage were flash frozen in liquid nitrogen, and subsequently stored at −80 °C until analyses.

### Analytical procedures and calculations

#### Proximate and mineral composition of feeds, whole fish and vertebrae

Moisture was measured by drying at 103 °C for 24 h, ash weighed after combustion at 540 °C and lipid after extraction with ethyl-acetate in fish tissue, and acid-extraction in the fish feed (Lie, 1991). Nitrogen was measured with a nitrogen analyzer (Vario Macro Cube, CN; Elementar Analysensysteme GmbH, Hanau, Germany) according to AOAC official methods of analysis (Sweeney & Rexroad, 1987), and protein calculated as N x 6.25. Starch was measured as glucose after enzymatic degradation, as described by (Hemre et al., 1989). A multi-element determination in the feed and tissue samples was done by ICP-MS (inductively coupled plasma mass spectrometry) (Julshamn et al., 1999). HPLC based method was used for determination of vitamin D_3_ (CEN, 2009).

### Apparent availability and retention of minerals

The apparent availability of target minerals was determined by using an inert marker namely yttrium oxide in the feed incorporated at 0.05%. Faeces were collected by stripping from the fish used for pooled whole fish samples (10 fish per tank). The feed and the faeces samples were analyzed for the trace minerals and yttrium according to the method mentioned above. The apparent availability coefficient (AAC) and retention were calculated based on the formulae presented in Hemre et al. (2016a).

### TRAP and ALP assay

On the day of analysis, vertebrae samples (from 4 fish per tank) were transferred to liquid nitrogen and crushed using a mortar and pestle. Following which, 300 mg of ground bone tissue was dissolved in 3 ml 0.9% w/v NaCl (154 mM) and homogenized on ice using a rod mixer (Polytron PT2100^®^, 5 bursts; 10 s/burst). From the obtained solution, 100 µL was transferred to 1.5 ml Eppendorf^®^ tubes and either 1 ml of a freshly prepared ALP buffer (100 mM NaCl, 5 mM MgCl_2_, 0.2 CaCl_2_, 0.1 mM ZnCl_2_, 25 mM glycine, 10 mM *p*-nitrophenyl phosphate, pH 10.2), or 1 ml of freshly prepared TRAP buffer (0.1 M NaCH_3_COO, 20 mM C_4_H_4_KNaO_6_ ⋅4H_2_O, 10 mM *p*-nitrophenyl phosphate, pH 5.3) were added. Both ALP and TRAP assays were performed in three analytical replicates. The reaction tubes were placed in a shaking incubator (25 °C, 200 rpm), in the dark. After 1 h, the reaction was stopped by adding 500 µL of a 1 M NaOH solution and the tubes were centrifuged (10 min, 12.000 g). The supernatant (140 µL) after centrifugation was measured for absorbance at 405 nm (Labsystems iEMS Reader MF^®^, Helsinki, Finland).

### Data analysis

In accordance with the design of the experiment, all the data were subjected to regression analysis, both linear and non-linear regression were performed to identify the best fit model (better R-square) to represent the data. In all regressions, analyzed dietary trace mineral concentration in the feeds NP0-NP400 was the independent variable (*X*-axis) and respective response criteria was the dependent (Y) variable. The linear regression was also tested for a significant deviation of the slope from zero (*P*-value, <0.05). When the non-linear regression was found to fit the data better, three models were compared, (i) BL1, broken-line with plateau; (ii) BL2, broken-line with two lines (Robbins, Saxton & Southern, 2006); and (iii) QP, quadratic plateau (Simongiovanni et al., 2012). The best fit model was used to estimate the minimum dietary levels required (along with 95% confidence intervals) for targeted micro-nutrients based on different response criteria.

## Results

Performance of Atlantic salmon parr and post-smolt during the experimental feeding period has been reported in detail by Hemre et al. (2016a). In brief, Atlantic salmon parr in Trial 1 more than tripled their body weight from an initial weight of 18.3 ± 2.2 g to a range of 78.6 ± 1.9 g to 87.3 ± 4.5 g. In Trial 2, initial post smolt weight was 228 ± 4.2 g and average final weight was 482 ± 17 g. Fish growth increased with increasing dietary NP in Trial 1 (parr); whereas, it was unaffected in Trial 2 (post-smolt). Survival was close to 100% in both trials, and with no difference between diet groups.

**Figure 1:**
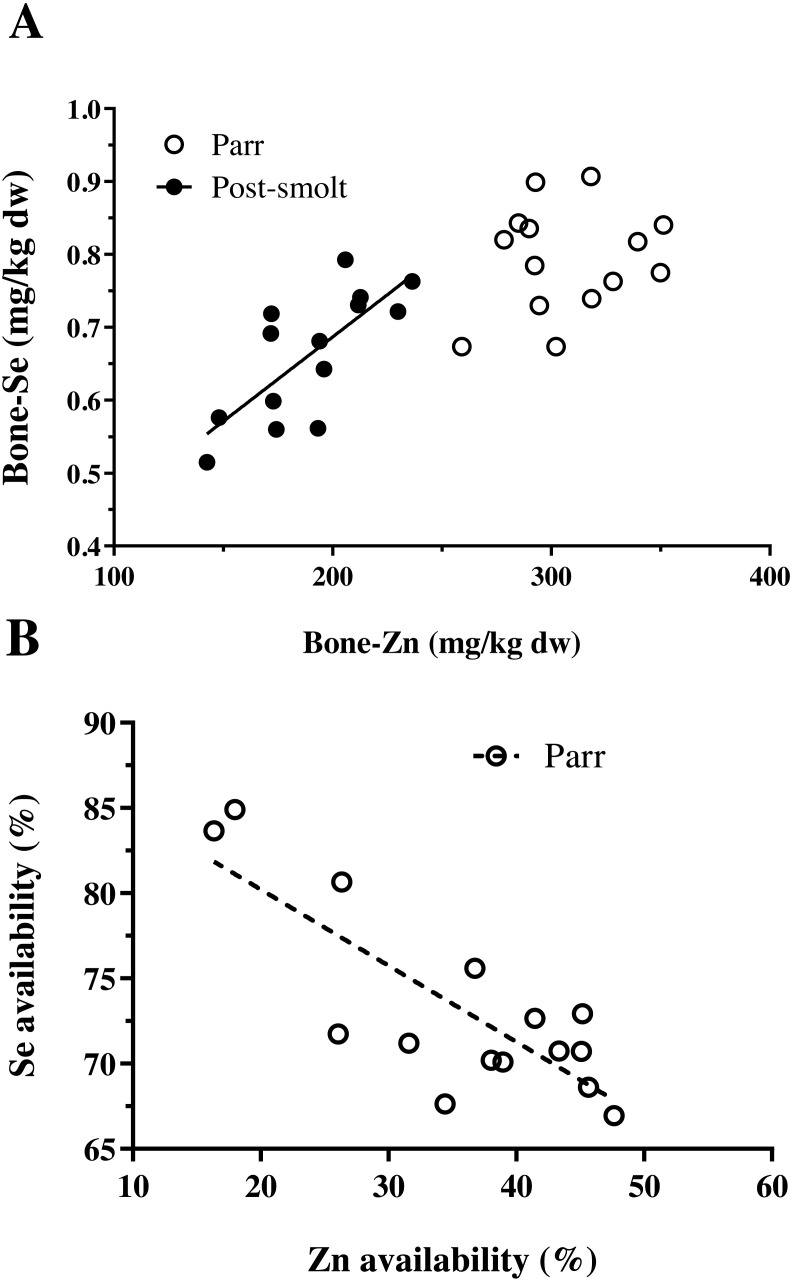
Interactions between Zn and Se in Atlantic salmon parr and post-smolt. (A) Relation between vertebral concentration of Zn and Se was found to be linear and positively correlated (*R*^2^, 0.55; Pearson r, 0.74; *p* = 0.002) in post-smolt (dark circles, solid line) but not in parr (open circles); (B) Relation between apparent availability of Zn and Se was linear, negatively correlated (*R*^2^, 0.65; Pearson r, −0.81; *p* = 0.0003) in Atlantic salmon parr (open circles, dotted line); data not available for post-smolt.

The concentration of trace minerals in the whole-body of Atlantic salmon are presented in Table 3. The concentration of Zn and Co in the whole-body were influenced by the graded inclusion of NP, in both the trials. The relation was linear for Zn and Co in both trials. The whole-body Se levels also increased linearly with NP inclusion, but data on the whole-body Se concentration and retention were already presented in Hamre et al. (2016). The concentration of iodine in the whole-body exhibited a non-linear relation with increasing inclusion of the NP. The whole-body concentrations of Cu, Mn and Fe were not affected by the inclusion of the NP. In addition to the whole-body, vertebral mineral concentrations were analyzed, the results of which are summarized in Table 4. In Trial 1, none of the analyzed trace minerals were significantly affected by a graded inclusion of the NP. In Trial 2, significant increase in vertebral concentration of Zn, Se and Co was observed with increasing NP inclusion. The relation was linear for Co; while Zn and Se showed respective non-linear relations. A positive and significant correlation between the concentration of Zn and Se in vertebrae of Atlantic salmon was observed in Trial 2, but not in Trial 1 (Fig. 1A).

**Table 3:** Whole body mineral composition of Atlantic salmon in Trial 1 (parr) and Trial 2 (post- smolt). Homogenized pooled samples of 10 fish per tank, 2–3 tanks per diet group. Data are presented as mean and pooled SD, in mg kg^−1^ wet weight. The column called “Regression” gives *R*^2^ and *p*-values for linear (L) or non-linear (N) regression models, whichever provided a significantly better fit to the data.

	**0 NP**	**25 NP**	**50 NP**	**100 NP**	**150 NP**	**200 NP**	**400 NP**	**Pooled SD**	**Regression**
**Trial 1 (freshwater, parr)**									
Co	–	–	–	0.025	0.033	0.037	0.065	0.003	^a^*R*^2^ = 0.93, L, *p* < 0.0001
Cu	1.10	1.10	1.10	1.20	1.20	1.20	1.25	0.08	n.s.
Fe	19.0	20.5	20.0	19.3	20.0	18.5	18.5	2.49	n.s.
Mn	2.30	2.50	2.30	2.33	2.40	2.00	1.50	0.50	n.s.
Zn	19.0	18.0	19.0	25.0	21.0	23.0	30.5	3.1	*R*^2^ = 0.42, L, *p* < 0.01
I	0.05	0.06	0.08	0.10	0.11	0.11	0.16	0.01	*R*^2^ = 0.85, N
**Trial 2 (seawater, post-smolt)**									
Co	0.005	0.008	0.01	0.016	0.024	0.028	0.041	0.002	*R*^2^ = 0.94, L, *p* < 0.0001
Cu	1.95	2.05	1.95	2.13	2.1	1.85	1.95	0.1	n.s.
Fe	12	12.5	12.5	12.3	12	12.5	12.5	0.3	n.s.
Mn	1.85	1.7	2.05	1.5	1.95	1.6	1.4	0.1	n.s.
Zn	22	21	21	22	27	29	28.5	1.5	*R*^2^ = 0.61, L, *p* < 0.001

**Notes.**

a using data only from 4 groups. NP, Nutrient package; n.s., not significant; Initial levels before the start (analyzed on triplicate samples, each consisting of 5 pooled fish, mean with SD in parenthesis, mg/kg wet weight): Trial 1: Mn, 1.6 (0.2); Fe, 20.0 (2.6); Co, <0.02; Cu, 0.9 (0.1); Zn, 36 (6). Trial 2 (mg/kg wet weight, analyzed on one pooled sample consisting of 15 fish): Mn, 2.3; Fe, 17; Co, 0.016; Cu, 2.1; Zn, 41. Data on whole body Se concentration from these trials have already been presented by Hamre et al. (2016).

**Table 4:** Vertebrae mineral composition of Atlantic salmon in trial 1 (parr) and trial 2 (post- smolt). Homogenized pooled samples of 10 fish per tank, 2–3 tanks per diet group. Data are presented as mean and pooled SD, in mg kg^−1^ dry weight. The column called “Regression” gives *R*^2^ and *p*-values for linear or non-linear regression models, whichever provided a significantly better fit to the data; unless otherwise specified with (N), the results are from linear regression.

	**0 NP**	**25 NP**	**50 NP**	**100 NP**	**150 NP**	**200 NP**	**400 NP**	**Pooled SD**	**Regression**
**Trial 1 (freshwater, parr)**									
Co	0.05	0.07	0.04	0.07	0.05	0.06	0.05	0.02	n.s.
Cu	1.89	1.94	1.78	1.96	1.74	1.52	2.1	0.2	n.s.
Fe	29.0	25.5	22.8	27.2	22.9	22.9	45.4	6.4	n.s.
Mn	71.1	73.6	77.3	66.8	71.9	63.5	63.9	2.5	n.s.
Se	0.79	0.83	0.77	0.79	0.79	0.67	0.84	0.04	n.s.
Zn	297.5	345.5	286.5	303.5	301.7	259.1	334.1	9.9	n.s.
**Trial 2 (seawater, post-smolt)**									
Co	0.02	0.03	0.04	0.08	0.1	0.13	0.19	0.005	*R*^2^ = 0.96, L, *p* < 0.0001
Cu	1.67	1.71	1.61	1.69	1.57	1.69	1.63	0.04	n.s.
Fe	38.8	39.3	37.7	64.1	41.1	37.9	36.6	11.9	n.s.
Mn	74.0	73.2	72.0	71.3	73.5	67.9	72.5	3.6	n.s.
Se	0.54	0.58	0.57	0.73	0.72	0.74	0.69	0.03	*R*^2^ = 0.81, N
Zn	142.5	173.6	170.6	190.6	192.2	233.2	203.9	10.4	*R*^2^ = 0.6, N

**Notes.**

NP Nutrient package n.s not significant

The macro-mineral composition of the bones were not affected by the nutrient package; mean (SD) across all the groups on their concentrations were as follows (mg kg^−1^ dry weight), trial 1: P, 102.3 (3); Ca, 203 (8); Ca:P ratio, 2 (0.01); Mg, 2.8 (0.1); Na, 11.5 (0.3) and K, 8.9 (0.6); trial 2: P, 100.4 (3); Ca, 201.5 (7.2); Ca:P ratio, 2.01 (0.01); Mg, 2.8 (0.1); Na, 11.2 (0.2) and K, 8 (0.3).

The data on the apparent availability and retention of trace minerals studied are presented in Table 5. Among the five trace minerals for which apparent availability coefficients (AAC, %) were measured, except for Co (linear relation), all the other four namely Cu, Mn, Zn and Se followed a non-linear pattern with increasing NP inclusion. A negative correlation between the AAC of Zn and Se was observed (Fig. 1B). Moreover, data on AAC from Trial 2 could not be obtained due to defective labeling of fecal samples from this trial. Retention of Cu and Mn decreased linearly; whereas, the retention of Zn exhibited a non-linear response with increasing NP inclusion in both the trials. Retention of Zn decreased at high NP inclusion in freshwater paar, but the contrary was observed in post-smolt salmon in seawater. Further, the retention values observed in post-smolt were lower than compared to Zn retention in parr.

**Figure 2:**
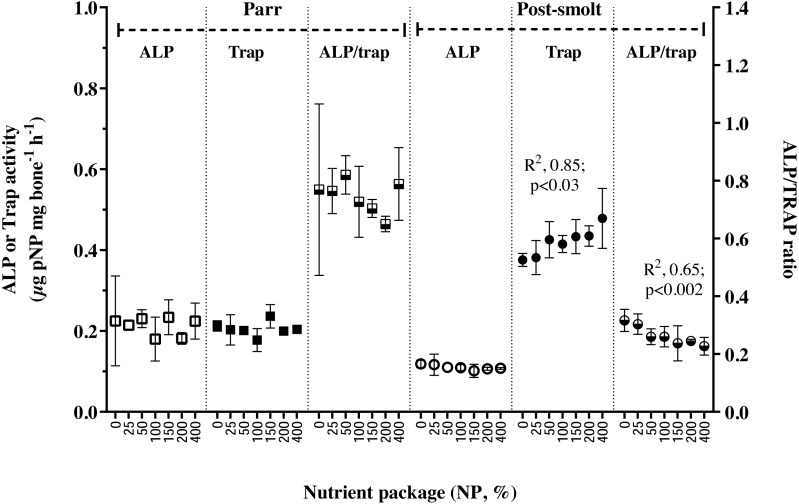
Markers of bone metabolism in vertebrae of Atlantic salmon parr and post-smolt fed low fish meal diets with graded inclusion of a multi-nutrient package. Activities of alkaline phosphatase (ALP, left Y-axis) and tartrate resistant acid phosphatase (TRAP, left Y-axis) and their ratio (ALP/TRAP, right Y-axis) in Atlantic salmon vertebrae samples from trial 1 (parr, square) and trial 2 (post-smolt, circles). In each trial, the groups are represented as follows, white symbols, ALP; dark symbols, TRAP and mixed, ALP/TRAP ratio. The activities are presented as µg pNP mg^−1^ bone h^−1^ (NP, para-nitrophenylphosphate), mean ± standard deviation of 2 groups, except for NP 100, 3 groups); in each group, samples from four individual fish were analyzed.

The activities ALP, TRAP and their ratio in the vertebral tissue were found not to be influenced by NP inclusion in Trial 1; on the other hand, in Trial 2, while APL activity remained stable, TRAP activity increased linearly with NP inclusion, thereby also resulting in a concomitant linear decline in the ALP/TRAP ratio (Fig. 2). Retention of studied trace minerals (Table 5) as percentage of intake decreased with increasing inclusion of NP in Trial 1, except for Se; the relation was found to be linear for Cu and Mn; non-linear for Zn. In Trial 2, retention of Cu and Mn was reduced with increasing NP, similar to the response in Trial 1, but the relation was non-linear for Mn in this trial. Regarding Zn, the response in trial 2 was contrary to that observed in Trial 1; the retention increased with increasing inclusion of NP and reached a plateau. The whole-body level of vitamin D3 is presented in Table 6. The whole-body status of vitamin D3 was not affected by inclusion of NP in Trial 1; whereas in Trial 2, a non-liner relation was seen in the whole-body vitamin D3 concentration with initial increase and reaching a plateau at 0.08 mg per kg diet.

**Table 5:** Apparent availability of micro-minerals in trial 1 (parr) and retention in trial 1 (parr) and trial 2 (post-smolt). Avilability calculations are based on pooled faeces samples from 10 fish per tank, while nutrient retention calculations are based on analysis of pooled samples of 10 whole fish per tank at the final sampling and of 15 fish as initial samples. The column “Regression” gives *R*^2^ and *p*-values for linear or non-linear regression models, whichever provided a significantly better fit to the data; unless otherwise specified with (N), the results are from linear regression.

		**0 NP**	**25 NP**	**50 NP**	**100 NP**	**150 NP**	**200 NP**	**400 NP**	**Pooled SD**	**Regression**
**Apparent availability (%)^b^**										
**Trial 1**	Co	-^a^	-^a^	-^a^	21.7	24.9	6.8	−2.5	5.9	*R*^2^ = 0.73, L, *p* < 0.01
	Cu	24.9	19.6	28.2	28.2	24.0	16.2	8.7	4.4	*R*^2^ = 0.64, N
	Mn	22.4	22.1	20.6	21.8	28.4	4.0	11.2	5.0	*R*^2^ = 0.79, N
	Se	70.4	69.1	74.1	70.4	69.4	76.2	84.3	2.3	*R*^2^ = 0.77, N
	Zn	41.1	39.6	39.1	41.6	41.9	26.2	17.2	4.4	*R*^2^ = 0.73, N
**Retention (%)**										
**Trial 1**	Cu	16.4	15.8	14.0	12.5	9.3	8.2	5.6	0.6	*R*^2^ = 0.86, L, *p* < 0.001
	Mn	8.0	8.2	7.4	6.7	5.0	4.6	1.9	2.5	*R*^2^ = 0.43, L, *p* < 0.01
	Zn	24.2	21.0	20.7	25.2	14.5	14.5	14.6	2.5	*R*^2^ = 0.53, N
**Trial 2**	Cu	20.3	18.8	16.3	16.1	15.1	12	8.1	0.3	*R*^2^ = 0.94, L, *p* < 0.001
	Mn	3.7	2.9	4.3	1.2	2.6	1.2	0.5	0.6	*R*^2^ = 0.60, L, *p* < 0.001
	Zn	3.13	0.75	−0.4	2.5	10.1	9.9	9.7	1.1	*R*^2^ = 0.67, N

**Notes.**

a Data not available due to levels being below the limit of quantification in the feed (for availability calculations), regression made using available data from 4 groups (100 NP-400 NP). Retention of Co could not be calculated due to the concentration in initial whole fish samples being below the limit of quantification.

b Apparent availability measurements could not be obtained for trial 2 due to unexpected labeling issues in marking of feces samples. Data on whole body Se retention from these trials have already been presented by Hamre et al. (2016).

Estimates from this study on dietary trace mineral and vitamin D3 levels required to meet the requirement of Atlantic salmon parr and post-smolt are summarized in Table 7. The dietary levels required in mg/kg diet (95% confidence intervals given in parenthesis) in low-fish meal practical diets for (i) Atlantic salmon parr are Cu, 12.7 ± 7.7 (very wide) and 11.2 ± 1.8 (7.1–15.2) based on whole-body saturation and AAC, respectively; Mn, 59 ± 6.5 (44.1–74), based on AAC; Zn, 110.8 ± 26 (53.1–168.5), based on AAC; iodine, 0.83 ±0.16 (0.3–1.6), based on whole-body saturation (ii) Atlantic salmon post-smolt: Cu, 13.4 ± 1.7 (9.7–17.1) based on whole-body saturation; Se, 0.79 ± 0.11 (0.63–0.93), based on vertebrae Se concentration; Zn, 158 ± 17 (120–197), based on retention; vitamin D3, 0.08 ± 0.01 (0.05–0.11), based on whole-body saturation. The corresponding available levels of the estimates obtained in trial 1 for Cu, Mn and Zn were within the range of the requirement reported for Atlantic salmon parr by NRC (2011). In trial 2, the requirement for post-smolt Atlantic salmon seemed to be similar in range with parr for Cu; whereas for Se, Zn and vitamin D3, the requirement estimate obtained were 2- to 4-fold higher than those recommended for Atlantic salmon parr or rainbow trout by NRC (2011).

## Discussion

The whole-body levels of most essential trace minerals are maintained within a compatible range for optimum growth and function (Lall, 2002). Fish have the ability to absorb dissolved minerals from water; however, quantitatively, the diet forms the major source to satisfy the requirements (Bury, Walker & Glover, 2003), while entero-hepatic and renal systems play major roles in essential trace mineral homeostasis (Hambidge, 2003; Wood, 2011). Gastrointestinal absorption is the major site of regulation for Fe, Cu, Mn and Zn, along with hepato-biliary excretion, except for Fe; whereas for Se and iodine, the major site of regulation is the kidney (Hambidge, 2003). Nutritive trace mineral uptake is actively regulated at the site of intestinal absorption and is highly influenced by interactions between other dietary components (Bury, Walker & Glover, 2003). The reduction in the AAC of Cu, Mn and Zn with highest NP inclusion in groups 200% and 400%; and inverse correlation between Zn and Se AAC values were in accordance with it. The requirement estimate (mean ± SE, mg/kg) obtained for Atlantic salmon parr in freshwater based on AAC as criteria were Cu, 2.8 ± 0.5; Mn, 13.6 ± 1.5 and Zn, 45.4 ± 10.7; the corresponding NRC (2011) recommendations for Atlantic salmon are Cu, 5; Mn, 10; and Zn, 37. In this regard, the requirement estimates (on available basis) for Cu, Mn and Zn obtained using the response of AAC were in agreement with the estimates presently recommended by NRC (2011). Despite intestinal trace mineral uptake being a well-regulated process, AAC as a criterion to determine requirement can be less reliable, especially with micro-minerals due to the entero-hepato-biliary circulation and associated endogenous excretions through the faeces. Therefore, whole-body concentration or retention, a relatively well-accepted approach to determine mineral requirement was also used (Shearer & Asgard, 1990; Shearer, 1995). Reduction in whole-body retention of Cu, Mn and Zn at higher NP (200 and 400%) levels confirmed the response of AAC, indicating that the requirement for Cu, Mn and Zn had been met with 100–150% NP levels for Atlantic salmon parr in freshwater, which are in agreement with recent reports in rainbow trout fed low fish meal diets (Read et al., 2014; Welker et al., 2016; Antony Jesu Prabhu et al., 2018a; Welker et al., 2018). Iodine requirement was met only at NP inclusion of 150–200%, indicating a requirement of 0.83 ± 0.16 mg iodine per kg diet for Atlantic salmon parr in freshwater, which is slightly lower but in close agreement (1-1.1 mg/kg diet) with the requirement reported in other salmonids, such as rainbow trout and Pacific salmon, *Oncorhynchus spp* (NRC, 2011).

**Table 6:** Vitamin D3 status in Atlantic salmon from trial 1 (parr) and trial 2 (post-smolt). Analyzed on homogenized pooled samples of 10 fish per tank, 2–3 tanks per diet group. Data are presented as mean ± pooled sd. The column called “Regression” gives *R*^2^ and *p*-values for linear or non-linear regression models, whichever provided a significantly better fit to the data; unless otherwise specified with (N), the results are from linear regression.

	**0 NP**	**25 NP**	**50 NP**	**100 NP**	**150 NP**	**200 NP**	**400 NP**	**Pooled SD**	**Regression^a^**
**Trial 1 (Freshwater, Parr)**									
Vit. D_3_	0.11	0.09	0.09	0.08	0.09	0.1	0.12	0.017	n.s.
**Trial 2 (Seawater, post-smolt)**									
Vit. D_3_	0.16	0.16	0.16	0.19	0.2	0.19	0.2	0.01	*R*^2^ = 0.69 (N)

**Notes.**

Initial levels before the start of the trial (analyzed on triplicate samples, each consisting of 5 pooled fish), mean with SD in parenthesis: Trial 1, 0.05 (0.00); Trial 2, one pooled sample.

**Table 7:** Estimates on dietary inclusion level recommendations to Atlantic salmon parr and post- smolt for micro-minerals and vitamin D3. The estimated requirement was obtained following non-linear regression and are presented as mean ± SE, along with regression coefficient (*R*^2^) and 95% confidence intervals (CL) wherever possible.

		**Parr**				**Post-smolt**				
**Mineral**	**Criterion**	**Estimated dietary level**	**95% CL**	*R*^**2**^	**available basis**	**Estimated dietary level**	**95% CL**	*R*^**2**^	**available basis^a^**	**NRC (2011)**
Cu	Whole body	12.7 ± 7.7	–	0.3	3.2 ± 1.9	13.4 ± 1.7	9.7–17.1	0.47		5
	Availability	11.2 ± 1.8	7.1–15.2	0.64	2.8 ± 0.5					
Mn	Availability	59 ± 6.5	44.1–74	0.8	13.6 ± 1.5					10
Se	Vertebrae					0.79 ± 0.11	0.63–0.93	0.81		0.15 (R.T)
Zn	Retention					158 ± 17	120–197	0.67		37
	Availability	110.8 ± 26	53.1–168.5	0.73	45.4 ± 10.7					
I	Whole body	0.83 ± 0.16	0.3–1.6	0.77	–					1.1 (R.T), 1 (P.S.)
Vit D_3_	Whole body					0.08 ± 0.01	0.05–0.11	0.69		0.04 (R.T)

**Notes.**

Three models (BL1, BL2 or QP) were compared and the requirement estimate generated from the best-fit model (higher *R*^2^) is presented in the table. BL1, broken-line with plateau; BL2, broken line with 2 slopes; and QP, quadratic plateau model. The estimate obtained were converted to available basis using the mean of apparent availability coefficient of NP0 to NP150 and compared with the recommendation reported in NRC (2011). R.T, rainbow trout; P.S, Pacific salmon.

a not possible to calculate due to lack of apparent availability data.

The response of post-smolt Atlantic salmon in seawater (Trial 2) showed that, whole-body Cu retention and the corresponding estimate of dietary Cu to satisfy the requirement was similar to parr in freshwater. Regarding Zn, the dietary level required to meet the requirement seemed to be higher in post-smolt than that obtained in parr. The overall retention of Zn was very low in post-smolt, ranging from -0.4 to 10.1%, in comparison to the retention in parr, 14.5 to 25.2%. Moreover, in Atlantic salmon parr, the retention decreased beyond 150 NP, the inclusion level at which the Zn requirement was estimated to be satisfied. The contrary was observed in seawater salmon with significantly higher retention at higher NP inclusion (150–400% NP). In the seawater trial, although the total Zn concentration ranged from 57.5 to 191 mg/kg diet, the low retention values raise concern over the availability. As mentioned earlier, the apparent availability coefficient could not be measured in the seawater trial, and it is possible that availability of dietary Zn to Atlantic salmon in seawater could be lower compared to freshwater. One hypothesis is that drinking of seawater can alter the ionic composition of the gut luminal contents, thereby altering the speciation and further availability of dietary Zn forms (Antony Jesu Prabhu et al., 2018b). In the event of availability not being lower in seawater Atlantic salmon, the possibility of an increased requirement for Zn towards maintenance or endogenous loss in seawater requires attention. The initial and final whole-body Zn concentrations were similar between Atlantic salmon parr and post-smolt studied here, but vertebrae Zn concentration in post-smolt was only half when compared to parr. Further, vertebrae Zn in freshwater parr was not responsive to increasing NP inclusion levels, but in post-smolt a linear increase in Zn concentration was observed with increasing NP of 0 to 400%. Vertebrae in fish is the major site of Zn storage (Satoh et al., 1987; Satoh, Takeuchi & Watanabe, 1987; Maage, Julshamn & Berge, 2001). In mammals, mobilization from bone may supply Zn to the soft tissues in periods of sub-optimal or deficient supply of dietary Zn (Brown, Chan & Smith, 1978); and depletion of vertebral Zn reserves in fish is a robust marker of dietary Zn deficiency or inadequacy (Ogino & Yang, 1978; Antony Jesu Prabhu, Schrama & Kaushik, 2016). It is therefore indicating that the supply of available Zn to post-smolt Atlantic salmon in seawater was not enough to maintain the saturation of Zn in the vertebrae.

In fish, mobilization of minerals from hard structures occurs during deficiency (Metz et al., 2014); during specific life stages of higher metabolic demand for certain minerals (Carragher & Sumpter, 1991; Baeverfjord et al., in press) or in response to hormonal imbalances (Mugiya & Watabe, 1977; Sbaihi et al., 2007; Sbaihi et al., 2009). The balance between mineralization and resorption of bone matrix is achieved through alkaline and acid phosphatase activities in osteoblasts and osteoclasts, respectively (Witten, 1997). Resorption and remodeling of extracellular matrix by osteoclasts are prerequisites for bone formation. Alkaline phosphatase (ALP) and tartrate-resistant acid phosphatase (TRAP) in the vertebrae of Atlantic salmon parr in freshwater were balanced and the ratio was near unity. In post-smolt A. salmon, however, 2-fold higher TRAP activity and reduced ALP/TRAP ratio to range from 0.2 to 0.4 suggested that bone resorption exceeded mineralization (Fjelldal et al., 2012a). Dietary limitation of available phosphorus along with continuous light regime was reported to increase TRAP activity and bone resorption in Atlantic salmon post-smolt (Fjelldal et al., 2012b; Fjelldal et al., 2016). Given the non-limiting supply of dietary P, and vertebral concentration of P, Ca or P:Ca ratio in A. salmon parr and post-smolt being similar, the increased TRAP activity in post-smolt is suggested to be triggered by the high dietary P:Ca ratio of nearly 3:1, resulting in low vertebral Zn status (Porn-Ngam et al., 1993; Hadley, Newman & Hunt, 2010). Targeted Zn release from bone during periods of dietary Zn inadequacy can occur; and can be independent of Ca and P de-mineralization in the bone (Brown, Chan & Smith, 1978). In rainbow trout, high P:Ca ratio did not affect the Ca, P or Ca:P ratio of the vertebrae, however, reduced the vertebral Zn and Mn levels; which was restored when the P:Ca ratio was maintained at 1:1 (Porn-Ngam et al., 1993).

The concentration of other analyzed trace minerals in the vertebrae of Atlantic salmon were similar in freshwater and seawater, except for Se and Co. The concentration of Se was lower in the vertebrae of seawater A. salmon and had a significant positive correlation with vertebrae Zn concentration; whereas no such relation was found in A. salmon during the freshwater phase. Vertebrae Co concentration was non-responsive to NP inclusion in A. salmon in freshwater, while it increased linearly with increasing NP inclusion in seawater reared post-smolt A. salmon, with two to four-fold higher concentration compared to A. salmon parr in freshwater. The differential response of vertebrae Co in the two trials could be related to the Zn status of the vertebrae, as it has been shown that Zn depleted conditions favor the replacement of Zn by Co in biological systems (Xu et al., 2007). Increasing nutrient premix inclusion did not result in increased whole-body or vertebrae concentrations of Cu, Fe and Mn. In rainbow trout juveniles, a similar response has been reported regarding these three trace minerals indicating that the requirement was met by the levels present in the non-supplemented diet (Antony Jesu Prabhu et al., 2018a). Fish meal is generally considered to be a good source of trace minerals (Julshamn, Haugsnes & Utne, 1978); however, plant ingredients contain equally or even more levels of Cu, Mn and Fe (NRC, 2011; Antony Jesu Prabhu et al., 2018a). The limiting trace minerals in plant ingredients are Zn and Se, for which a significant linear or non-linear response was recorded in whole-body or tissue levels with increasing NP inclusion in A. salmon parr in freshwater; and with both whole-body and vertebrae concentration in A. salmon post-smolt in seawater. Selenium requirement has not been tested in Atlantic salmon (NRC, 2011). Data available on rainbow trout indicates the requirement to range from 0.15 to 0.38 mg/kg diet (Hilton, Hodson & Slinger, 1980; NRC, 2011). In this study, it was not possible to estimate the dietary level required for Se with any of the analysed criteria in A. salmon parr; whereas in post-smolt A. salmon, the total Se level required in the diet was estimated to range between 0.63 to 0.93 mg/kg diet based on vertebrae Se as the criterion. Whole-body Se concentration had a linear response to increasing NP inclusion; while Se retention was unaffected (Hamre et al., 2016) The estimate on the dietary Se level required for post-smolt Atlantic salmon in seawater was similar to the values reported for other marine species eg. 0.8 mg/kg diet, cobia (Liu et al., 2010); 0.7–0.8 mg/kg diet, grouper (Lin & Shiau, 2005); but higher than the present maximum limit of 0.5 mg/kg total Se allowed in salmonid feeds in the EU ((EC) No 1831/2003 and amendments). The apparent availability of Se was high and ranged from 69 to 84% in A. salmon parr, with a significant negative correlation with the apparent availability coefficient of Zn. In the high NP diets (200 and 400%), when Zn availability reduced, Se availability increased; the correlation was not significant for others trace minerals. In zebrafish, a high Zn diet resulted in increased Se in the faeces (C Hogstrand, Pers. Comm., 2018); and mammalian Zn transporter (ZIP8) plays a major role in selenite (HSeO^3−^) uptake (McDermott et al., 2016). Therefore, competitive inhibition of Se uptake by Zn, and vice-versa are possible. In salmonid feeds, an inverse relation between Zn and Se availability opens a new challenge as both are limiting in plant ingredient-based feeds and the maximal limit of supplementation for Zn and Se are under strict scrutiny by the EU legislation ((EC) No 1831/2003 and amendments). Given that this study presents only a correlative indication, more data is required to better understand the relationship between Zn and Se availability in fish feeds, especially with high plant ingredient inclusion.

Vitamin D3 is known to modulate bone and trace mineral metabolism in fish and especially salmonids (Vielma et al., 1999). The requirement for vitamin D3 to fish can range from 0.006 to 0.125 mg per kg diet (250 to 5000 IU), depending on the species, life stage and rearing environment (Lock et al., 2010). Among salmonids, it is reported that rainbow trout and Atlantic salmon require 0.04 and 0.06 mg vitamin D3 per kg diet, respectively (Barnett, Cho & Slinger, 1982; Woodward, 1994). In Atlantic salmon, it has been suggested that the vitamin D endocrine system can be differentially regulated during adaptation to life in seawater. In the present study, the vitamin D3 levels of Atlantic salmon in seawater were higher than in freshwater. In response to increasing NP inclusion, whole-body vitamin D3 levels had a dose-dependent increase, as observed for tissue vitamin D3 status by Horvli, Aksnes & Lie (1998). Moreover, the vitamin D3 levels in the whole-body reached a saturation point at 0.08 ± 0.01 mg per kg diet; the 95% confidence intervals of which are within the range reported in fish, specifically salmonids (0.05–0.11 mg per kg diet). The dietary level of vitamin D3 required to attain whole-body saturation in the present study was 1.5 to 2-fold higher than reported earlier for Atlantic salmon (Woodward, 1994; Halver, 2002). It is not clear if the requirement for vitamin D3 in Atlantic salmon was higher in seawater than in freshwater, due to the lack of a clear dose–response in freshwater parr. However, considering that the earlier estimates available are from freshwater salmonids namely rainbow trout and Atlantic salmon parr in freshwater, the present study provides novel data on vitamin D3 in post-smolt Atlantic salmon.

## Conclusion

The NP approach was useful to identify ‘practical recommendations’ for dietary trace mineral and vitamin D3 levels in plant-based, low fish meal diets for Atlantic salmon parr and post-smolt. Atlantic salmon parr held in freshwater were able to satisfy the requirement for the trace minerals Zn, Mn, Se, Cu, and Fe through supply from 100-150 NP, corresponding to 101–132, 47–63, 0.6–0.8, 12–16 and 150–166 mg kg^−1^diet, respectively; for iodine, dietary supply from 150–200 NP corresponding to 0.7–1.6 mg kg^−1^diet was required. In the seawater, Atlantic salmon post-smolt required trace mineral and vitamin D3 levels as supplied through 150-200 NP, corresponding to 140–177, Zn; 61–67, Mn; 0.9–1, Se; and vitamin D3, 0.06–0.09 mg kg^−1^ diet to fulfil the requirement, except for Cu which was satisfied at 100–150 NP, equivalent to 13–14 mg kg^−1^ diet.

## Additional Information and Declarations

### Data Availability

The following information was supplied regarding data availability: All the raw data is available in the Tables.

## References

[ref-1] Antony Jesu Prabhu P, Schrama J, Fontagné-Dicharry S, Mariojouls C, Surget A, Bueno M, Geurden I, Kaushik SJ (2018a). Evaluating dietary supply of microminerals as a premix in a complete plant ingredient-based diet to juvenile rainbow trout (*Oncorhynchus mykiss*). Aquaculture Nutrition.

[ref-2] Antony Jesu Prabhu P, Schrama JW, Kaushik SJ (2016). Mineral requirements of fish: a systematic review. Reviews in Aquaculture.

[ref-3] Antony Jesu Prabhu P, Stewart T, Silva M, Amlund H, Ørnsrud R, Lock EJ, Waagbo R, Hogstrand C (2018b). Zinc uptake in fish intestinal epithelial model RTgutGC: impact of media ion composition and methionine chelation. Journal of Trace Elements in Medicine and Biology.

[ref-4] Baeverfjord G, Antony Jesu Prabhu P, Fjelldal PG, Albrektsen S, Hatlen B, Denstadli V, Ytteborg E, Takle H, Lock E-J, Berntssen MHG, Lundebye A-K, Åsgård T, Waagbø R Mineral nutrition and bone health in salmonids. Reviews in Aquaculture.

[ref-5] Barnett BJ, Cho CY, Slinger SJ (1982). Relative Biopotency of Dietary Ergocalciferol and Cholecalciferol and the Role of and Requirement for Vitamin D in Rainbow Trout (Salmo gairdneri). The Journal of nutrition.

[ref-6] Brown ED, Chan W, Smith JC (1978). Bone mineralization during a developing zinc deficiency. Proceedings of the Society for Experimental Biology and Medicine.

[ref-7] Bury NR, Walker PA, Glover CN (2003). Nutritive metal uptake in teleost fish. Journal of Experimental Biology.

[ref-8] Carragher JF, Sumpter JP (1991). The mobilization of calcium from calcified tissues of rainbow trout (Oncorhynchus mykiss) induced to synthesize vitellogenin. Comparative Biochemistry Physiology Part A: Physiology.

[ref-9] CEN (2009). Foodstuffs–Determination of vitamin D by high performance liquid chromatography Measurement of cholecalciferol (D3) or ergocalciferol (D2). Comite Europeen de Normalisation.

[ref-10] Fjelldal PG, Hansen T, Breck O, Ørnsrud R, Lock EJ, Waagbø R, Wargelius A, Witten Eckhard (2012a). Vertebral deformities in farmed Atlantic salmon (*Salmo salar* L.)—etiology and pathology. Journal of Applied Ichthyology.

[ref-11] Fjelldal P, Hansen T, Lock EJ, Wargelius A, Fraser T, Sambraus F, El-Mowafi A, Albrektsen S, Waagbø R, Ørnsrud RJAn (2016). Increased dietary phosphorous prevents vertebral deformities in triploid A tlantic salmon (*Salmo salar* L.). Aquaculture Nutrition.

[ref-12] Fjelldal PG, Lock EJ, Hansen T, Waagbø R, Wargelius A, Gil Martens L, El-Mowafi A, Ørnsrud R (2012b). Continuous light induces bone resorption and affects vertebral morphology in Atlantic salmon (*Salmo salar* L.) fed a phosphorous deficient diet. Aquaculture Nutrition.

[ref-13] Hadley KB, Newman SM, Hunt JR (2010). Dietary zinc reduces osteoclast resorption activities and increases markers of osteoblast differentiation, matrix maturation, and mineralization in the long bones of growing rats. The Journal of Nutritional Biochemistry.

[ref-14] Halver JE, Halver J, Hardy R (2002). The vitamins. Fish nutrition.

[ref-15] Hambidge M (2003). Biomarkers of trace mineral intake and status. The Journal of Nutrition.

[ref-16] Hamre K, Sissener NH, Lock E-J, Olsvik PA, Espe M, Torstensen BE, Silva J, Johansen J, Waagbø R, Hemre G-I (2016). Antioxidant nutrition in Atlantic salmon (*Salmo salar*) parr and post-smolt, fed diets with high inclusion of plant ingredients and graded levels of micronutrients and selected amino acids. PeerJ.

[ref-17] Hemre G-I, Lie Ø, Lied E, Lambertsen G (1989). Starch as an energy source in feed for cod (*Gadus morhua*): digestibility and retention. Aquaculture.

[ref-18] Hemre GI, Lock EJ, Hamre K, Epse M, Waagbø R, Fountoulaki E, Izquierdo M, Tocher DR, Kaushik SJ (2016b). Aquaculture nutrient requirements: understanding vitamins, minerals and other nutrtients in fish feed diets based on plant derived ingredients. ARRAINA Technical booklet. 16.

[ref-19] Hemre G-I, Lock E-J, Olsvik PA, Hamre K, Espe M, Torstensen BE, Silva J, Hansen A-C, Waagbø R, Johansen J (2016a). Atlantic salmon (*Salmo salar*) require increased dietary levels of B-vitamins when fed diets with high inclusion of plant based ingredients. PeerJ.

[ref-20] Hilton JW, Hodson PV, Slinger SJ (1980). The requirement and toxicity of selenium in rainbow trout (*Salmo gairdneri*). The Journal of nutrition.

[ref-21] Horvli O, Aksnes L, Lie Ø (1998). Tissue distribution of vitamin D3 in Atlantic salmon (*Salmo salar)*: effect of dietary level. Aquaculture Nutrition.

[ref-22] Julshamn K, Brenna J, Holland R, Tanner S (1999). Plasma source mass spectrometry–new developments and applications. Royal Society of Chemistry.

[ref-23] Julshamn K, Haugsnes J, Utne F (1978). The contents of 14 major and minor elements (minerals) in Norwegian fish species and fish byproducts, determined by atomic absorption spectrophotometry. Fiskeridirektoratets skrifter, Serie ernæring.

[ref-24] Kaushik S, Guillaume J, Kaushik S, Bergot P, Metailler R (2002). Mineral nutrition. Nutrition and feeding of fish and crustaceans.

[ref-25] Lall SP, John EH, Ronald WH (2002). The minerals. Fish nutrition (Third edition).

[ref-26] Lall SP, Lewis-McCrea LM (2007). Role of nutrients in skeletal metabolism and pathology in fish–an overview. Aquaculture.

[ref-27] Lie Ø (1991). Studies on digestion, deposition and fatty acid composition of lipids in cod (*Gadus Morhua*). PhD Thesis.

[ref-28] Lin YH, Shiau SY (2005). Dietary selenium requirements of juvenile grouper, Epinephelus malabaricus. Aquaculture.

[ref-29] Liu K, Wang XJ, Ai Q, Mai K, Zhang W (2010). Dietary selenium requirement for juvenile cobia, Rachycentron canadum L. Aquaculture Research.

[ref-30] Lock EJ, WaagbØ R, Bonga SWendelaar, Flik G (2010). The significance of vitamin D for fish: a review. Aquaculture Nutrition.

[ref-31] Lorentzen M, Maage A (1999). Trace element status of juvenile Atlantic salmon (*Salmo salar* L.) fed a fish-meal based diet with or without supplementation of zinc, iron, manganese and copper from first feeding. Aquaculture Nutrition.

[ref-32] Maage A, Julshamn K, Berge GE (2001). Zinc gluconate and zinc sulphate as dietary zinc sources for Atlantic salmon. Aquaculture Nutrition.

[ref-33] McDermott JR, Geng X, Jiang L, Gálvez-Peralta M, Chen F, Nebert DW, Liu Z (2016). Zinc- and bicarbonate-dependent ZIP8 transporter mediates selenite uptake. Oncotarget.

[ref-34] Metz JR, Leeuwis RHJ, Zethof J, Flik G (2014). Zebrafish (*Danio rerio*) in calcium-poor water mobilise calcium and phosphorus from scales. Journal of Applied Ichthyology.

[ref-35] Mugiya Y, Watabe N (1977). Studies on fish scale formation and resorption—II. Effect of estradiol on calcium homeostasis and skeletal tissue resorption in the goldfish, *Carassius auratus*, and the killifish, Fundulus heteroclitus. Comparative Biochemistry and Physiology Part A: Physiology.

[ref-36] NRC (2011). Nutrient requirements of fish and shrimp.

[ref-37] Ogino O, Yang GY (1978). Requirement of rainbow trout for dietary zinc. Bulletin of the Japanese Society of Scientific Fisheries.

[ref-38] Olesen I, Myhr AI, Rosendal GK (2011). Sustainable aquaculture: are we getting there? Ethical perspectives on salmon farming. Journal of Agricultural Environmental Ethics.

[ref-39] Porn-Ngam N, Satoh S, Takeuchi T, Watanabe T (1993). Effect of the ratio of phosphorus to calcium on zinc availability to rainbow trout in high phosphorus diet. Nippon Suisan Gakkaishi.

[ref-40] Read ES, Barrows FT, Gibson Gaylord T, Paterson J, Petersen MK, Sealey WM (2014). Investigation of the effects of dietary protein source on copper and zinc bioavailability in fishmeal and plant-based diets for rainbow trout. Aquaculture.

[ref-41] Robbins K, Saxton A, Southern L (2006). Estimation of nutrient requirements using broken-line regression analysis. Journal of Animal Science.

[ref-42] Satoh S, Tabata K, Izume K, Takeuchi T, Watanabe T (1987). Effect of dietary tricalcium phosphate on availability of zinc to rainbow trout. Nippon Suisan Gakkaishi.

[ref-43] Satoh S, Takeuchi T, Watanabe T (1987). Availability to Rainbow trout of zinc in white fish meal and of various zinc compounds. Nippon Suisan Gakkaishi.

[ref-44] Sbaihi M, Kacem A, Aroua S, Baloche S, Rousseau K, Lopez E, Meunier F, Dufour S (2007). Thyroid hormone-induced demineralisation of the vertebral skeleton of the eel, *Anguilla anguilla*. General and Comparative Endocrinology.

[ref-45] Sbaihi M, Rousseau K, Baloche S, Meunier F, Fouchereau-Peron M, Dufour S (2009). Cortisol mobilizes mineral stores from vertebral skeleton in the European eel: an ancestral origin for glucocorticoid-induced osteoporosis?. Journal of Endocrinology.

[ref-46] Shearer KD (1995). The use of factorial modeling to determine the dietary requirements for essential elements in fishes. Aquaculture.

[ref-47] Shearer KD, Asgard T (1990). Availability of dietary magnesium to rainbow trout as determined by apparent retention. Aquaculture.

[ref-48] Simongiovanni A, Corrent E, Le Floc’h N, Van Milgen J (2012). Estimation of the tryptophan requirement in piglets by meta-analysis. Animal.

[ref-49] Sweeney R, Rexroad P (1987). Comparison of LECO FP-228 “nitrogen determinator” with AOAC copper catalyst Kjeldahl method for crude protein. Journal-Association of Official Analytical Chemists.

[ref-50] Vielma J, Lall S, Koskela J, Mattila P (1999). Influence of low dietary cholecalciferol intake on phosphorus and trace element metabolism by rainbow trout (*Oncorhynchus mykiss*, Walbaum). Comparative Biochemistry and Physiology - Part A: Molecular & Integrative Physiology.

[ref-51] Watanabe T, Kiron V, Satoh S (1997). Trace minerals in fish nutrition. Aquaculture.

[ref-52] Welker T, Barrows F, Overturf K, Gaylord G, Sealey WJAn (2016). Optimizing zinc supplementation levels of rainbow trout (*Oncorhynchus mykiss*) fed practical type fishmeal-and plant-based diets. Aquaculture Nutrition.

[ref-53] Welker TL, Overturf K, Abernathy J, Barrows FT, Gaylord GJJotWAS (2018). Optimization of Dietary Manganese for Rainbow Trout, *Oncorhynchus mykiss*, Fed a Plant-based Diet. Journal of World Aquaculture Society.

[ref-54] Witten PE (1997). Enzyme histochemical characteristics of osteoblasts and mononucleated osteoclasts in a teleost fish with acellular bone (*Oreochromis niloticus*, Cichlidae). Journal of Cell and Tissue Research.

[ref-55] Wood CM, Wood CM, Farrell AP, Brauner CJ (2011). An introduction to metals in fish physiology and toxicology: basic principles. Fish physiology.

[ref-56] Woodward B (1994). Dietary vitamin requirements of cultured young fish, with emphasis on quantitative estimates for salmonids.. Aquaculture.

[ref-57] Xu Y, Tang D, Shaked Y, Morel F (2007). Zinc, cadmium, and cobalt interreplacement and relative use efficiencies in the coccolithophore *Emiliania huxleyi*. Limnology and Oceanography.

[ref-58] Ytrestøyl T, Aas TS, Åsgård T (2015). Utilisation of feed resources in production of Atlantic salmon (*Salmo salar*) in Norway. Aquaculture.

